# The Effect of the Intelligent Sepsis Management System on Outcomes among Patients with Sepsis and Septic Shock Diagnosed According to the Sepsis-3 Definition in the Emergency Department

**DOI:** 10.3390/jcm8111800

**Published:** 2019-10-27

**Authors:** Juhyun Song, Hanjin Cho, Dae Won Park, Sejoong Ahn, Joo Yeong Kim, Hyeri Seok, Jonghak Park, Sungwoo Moon

**Affiliations:** 1 Department of Emergency Medicine, Korea University Ansan Hospital, Jeokgeum-ro 123, Danwon-gu, Gyeonggi-do 15355, Korea; songcap97@hotmail.com (J.S.);; 2 Division of Infectious Diseases, Department of Internal Medicine, Korea University Ansan Hospital, Jeokgeum-ro 123, Danwon-gu, Gyeonggi-do 15355, Korea; 3 National Emergency Medical Center, National Medical Center, Eulji-ro 245, Jung-gu, Seoul 04564, Korea

**Keywords:** emergency department, mortality, sepsis, septic shock, Surviving Sepsis Campaign guidelines

## Abstract

We developed a novel computer program, the Intelligent Sepsis Management System, based on Sepsis-3 definitions and 2016 Surviving Sepsis Campaign guidelines and performed a quasi-experimental pre-post study to assess its effect on compliance with the Surviving Sepsis Campaign guidelines and outcomes in patients with sepsis and septic shock. During the pre-period, patients were managed with usual care. During the post-period, patients were managed using the Intelligent Sepsis Management System upon arrival at the emergency department. A total of 631 patients were enrolled (pre-period, 316; post-period, 315). The overall compliance with the Surviving Sepsis Campaign guidelines’ bundle improved (pre-period 10.8% vs. post-period 54.6%; *p* < 0.001). The post-period showed significantly lower 30-day mortality than the pre-period (pre-period 37.3% vs. post-period 29.5%; *p* = 0.037), but was not a protective factor for 30-day mortality, with an adjusted hazard ratio (95% confidence interval) of 0.75 (0.55–1.04) (*p* = 0.151). The associated factors for 30-day mortality were age, sequential organ failure assessment score, overall compliance, and lactate levels. The 30-day mortality was significantly lower in the compliance group than in the non-compliance group (27.2% vs. 36.5%; *p* = 0.002). After implementation of the Intelligent Sepsis Management System, overall compliance with the Surviving Sepsis Campaign guidelines improved and was associated with reduced 30-day mortality. However, we could not verify the causal effect of this system on 30-day mortality.

## 1. Introduction

Sepsis is a leading cause of mortality and long-term disability worldwide [1,2]; thus, it is a major public health concern accounting for increased health care costs [3]. The reported incidence of sepsis continues to increase [4], possibly reflecting improved recognition and an increase in the aging population with various comorbidities [5]. Early detection and appropriate treatment are essential to improving outcomes of sepsis [5,6].

Quality improvement programs have improved the process of sepsis care and survival outcomes [7,8,9,10,11,12] and electronic medical record surveillance systems have facilitated sepsis detection and improved outcomes [13,14,15]. A real-time sepsis alert system increased early therapeutic and diagnostic interventions among non-intensive care unit patients at risk for sepsis [16].

The 2016 Surviving Sepsis Campaign (SSC) guidelines recommend the timely implementation of key interventions such as antibiotics, fluids, microbiologic culture, and lactate measurements [5]. The latest Sepsis-3 definitions recommend using the quick sequential organ failure assessment (qSOFA) score for sepsis screening outside the intensive care unit (ICU) [16,17]. Here, we developed a novel computerized program, the Intelligent Sepsis Management System (i-SMS), according to the Sepsis-3 definitions and 2016 SSC guidelines. This study assessed the effect of the i-SMS on SSC guideline compliance and outcomes among sepsis and septic shock patients.

## 2. Materials and Methods

### 2.1. Study Design and Setting

This quasi-experimental pre-post study was conducted at the general Emergency Department (ED) of Korea University Ansan Hospital in South Korea.

### 2.2. Study Population

We included adult patients (age ≥18 years) with initial qSOFA scores ≥2 upon ED arrival and who met the diagnostic criteria for sepsis. We excluded patients with cardiac arrest on ED arrival and those who died within 2 h of ED presentation (exclusion criteria). In other words, the inclusion criteria are (1) meeting the diagnostic criteria for sepsis in accordance with the Sepsis-3 definitions, (2) adult patients (age ≥18 years), and (3) initial qSOFA scores ≥2 on ED arrival. Sepsis is now defined as a life-threatening organ dysfunction caused by a dysregulated host response to infection. After considering various test results (radiologic findings, laboratory results, microbiologic culture, and patient symptoms/signs), we comprehensively determined the presence of infection and diagnosed sepsis according to the latest Sepsis-3 definitions. Organ dysfunction was represented by a SOFA score increase to ≥2.

During the pre-period (1 January 2016–25 September 2017), sepsis patients were managed with usual care. In the pre-period, patients with initial qSOFA scores ≥2 were identified via a search for electronic medical records and were examined by retrospectively reviewing their medical records and test results. Patients meeting all inclusion criteria were included in the pre-period group. During the post-period (26 September 2017–30 January 2019), patients were managed using the i-SMS upon ED arrival. In the post-period group, sepsis patients were prospectively registered using the i-SMS. They were then examined through reviews of their medical records and test results. Patients meeting all inclusion criteria were included in the post-period group.

This study was conducted according to the principles of the Declaration of Helsinki and was approved by the Institutional Review Board of the hospital (number 2018AS0089). We obtained informed consent from eligible patients before enrollment.

### 2.3. Intervention

The i-SMS was implemented in September 2017 after a development period of eight months. The development team included two infectious disease (ID) specialists, three attending emergency physicians, two triage nurses, and one expert computer programmer. The i-SMS system was designed as follows. Upon ED arrival, triage nurses check patient vital signs and mental status. For patients with an initial qSOFA score ≥2, the digital Order Communication System automatically highlights their name in violet to increase visibility to the ED physicians. Such patients are also automatically recorded into a qSOFA-positive registry group. Patients with cardiac arrest on arrival are excluded by the i-SMS. ED physicians can directly activate the next step in the protocol, which prompts the user to specify the presence and location of infection in qSOFA-positive patients. This step is not routinely activated in most patients, as confirmation of infection requires medical history, physical examination, and laboratory or imaging results. If not triggered directly by the ED physician, the i-SMS automatically activates the second step 90 min after patient arrival and prompts the ED physician to either select the suspected infection site or to check “no infection”. If the latter is selected, the highlighted violet background color of the patient name reverts to the standard white, and the patient is recorded as qSOFA-positive, but sepsis-negative.

If the ED physician selects infection sites, thereby confirming the presence of an infection, a SOFA score calculation window is triggered as a third step. Simultaneously, a bundle set order appears in the physician order window. The complete bundle set order includes orders for microbiologic culture, lactate measurement, biomarker (procalcitonin and C-reactive protein (CRP)) measurement, and a text recommendation to use antibiotics and administer appropriate fluids. The physician can edit the bundle on a case-by-case basis as needed. Patients with SOFA scores ≥2 are registered as sepsis (and qSOFA)-positive, whereas patients with SOFA scores of 0 or 1 are recorded as qSOFA-positive, but sepsis-negative.

Finally, the i-SMS reminds ED physicians, via a pop-up window, to check whether their patients meet the criteria for septic shock and, if so, to initiate appropriate management. Prior to applying the i-SMS, three educational sessions on i-SMS were offered to the ED physicians.

### 2.4. Definitions

A 2016 Sepsis-3 definitions taskforce proposed a new definition according to which sepsis is a life-threatening organ dysfunction caused by a dysregulated host response to an infection [6]. Organ dysfunction can be represented by a SOFA score increase to ≥2. Septic shock is defined as a subset of sepsis in which particularly profound circulatory, cellular, and metabolic abnormalities are associated with greater risks of mortality than with sepsis alone [6,18]. Septic shock is clinically identified by a vasopressor requirement to maintain a mean arterial pressure ≥65 mmHg and a serum lactate level >2 mmol/L (>18 mg/dL) in the absence of hypovolemia. In out-of-hospital, ED, or general ward settings, adult patients with a suspected infection can be promptly identified if they have at least two of the following clinical criteria comprising the qSOFA score: respiratory rate ≥22/min, systolic blood pressure ≤100 mmHg, or altered mental status [6].

An independent ID specialist blinded to the pre- and post-period study group designations assessed SSC guideline compliance based on antibiotic and fluid administration, blood culture, and measurement of lactate levels. The 2016 SSC guidelines recommend that intravenous antibiotics be initiated as soon as possible after recognition and within one hour for sepsis and septic shock [5]. However, in the clinical setting, the definitive diagnosis of sepsis takes time. We set 2 h as the time required to diagnose sepsis; therefore, we considered the administration of broad-spectrum antibiotics within 3 h of ED presentation as bundle compliant. The guidelines recommend the administration of at least 30 mL/kg of intravenous (IV) crystalloid fluid within the first 3 h for the resuscitation of sepsis-induced hypoperfusion patients [5]. The guidelines also recommend blood cultures before initiating antibiotic therapy and guide resuscitation to normalize elevated lactate levels as a marker of tissue hypoperfusion [5]. Because a single lactate measurement might be insufficient, we considered at least two lactate measurements within 6 h of ED presentation to be bundle compliant.

### 2.5. Data Collection

The same variables were collected in both the pre- and post-periods as follows: patient demographics, vital signs and mental status, Korean triage acuity scale (KTAS), infection sites, Charlson comorbidity index score, severity, compliance with SSC guidelines, laboratory results, antibiotic therapy including time to administration of the first dose, pre-ED antibiotic treatment, length of hospital stay, length of ICU stay, and all-cause 7-, 14-, and 30-day mortality. Patients were followed-up until 30 days after ED presentation. If they were discharged or transferred to other institutions earlier than 30 days after ED presentation, we collected data by telephone conversation with patients, their legal representatives, or their physicians.

### 2.6. Outcomes 

The primary outcomes were 30-day all-cause mortality and compliance with the SSC guidelines’ bundle. The secondary outcome was time to initial antibiotic administration upon ED arrival.

### 2.7. Statistical Analyses 

We obtained descriptive statistics for all variables evaluated in the study population. For continuous variables, the means and standard deviations were used for normal distributions and medians and interquartile ranges (IQRs) for skewed distributions. Proportions (%) were used to describe categorical variables. The pre- and post-period groups were compared and statistical significance was assessed using Student’s *t*-test or Mann–Whitney *U*-test for quantitative variables according to their distributions. For categorical variables, Pearson’s χ^2^ or Fisher’s exact tests were performed according to expected frequencies in the contingency table. Risk factors for 30-day mortality were assessed using Cox proportional hazards models. All variables with *p* ≤ 0.1 in the univariate analysis were included in the multivariate analysis. The overall patient group, as well as the compliance and non-compliance groups, were compared. Kaplan–Meier curves were generated and log-rank tests were used to compare 30-day mortality. *p* < 0.05 was considered statistically significant.

All statistical analyses were performed using IBM SPSS Statistics for Windows, version 20.0 (IBM Corp., Armonk, NY, USA).

## 3. Results

**Figure 1:**
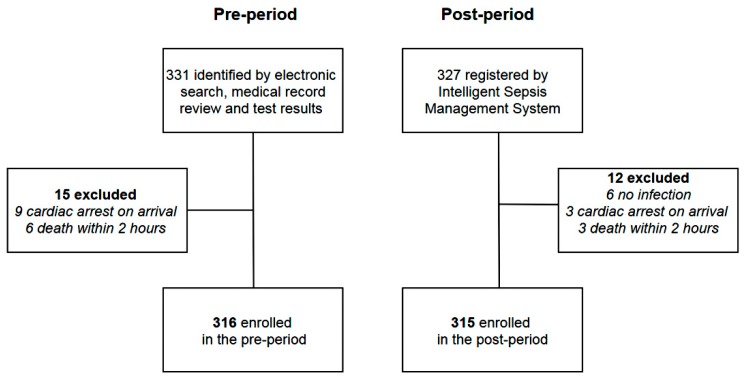
Flow chart of the study population.

During the pre-period, 810 patients were selected via an electronic search for subjects with initial qSOFA scores ≥2. Among them, 331 patients had sepsis following a retrospective review of the medical records and test results. Fifteen patients were excluded owing to cardiac arrest on arrival (*n* = 9) or death within two hours of ED presentation (*n* = 6). Therefore, a total of 316 patients were included in the pre-period group. During the post-period, 327 patients were registered using i-SMS. Among them, 12 patients were excluded after reviewing the medical records and test results owing to lack of evidence of infection (*n* = 6), cardiac arrest on arrival (*n* = 3), or death within two hours of ED presentation (*n* = 3). Thus, a total of 315 patients were included in the post-period group. Figure 1 shows the flowchart of the study population.

### 3.1. Characteristics of Pre- and Post-Implementation

The baseline characteristics of the study population are summarized in Table 1. We compared the pre-period and post-period groups. The median ages of patients in the pre- and post-periods were 74 (IQR, 64–81) and 77 (IQR, 65–83) years, respectively (*p* = 0.048). The median procalcitonin levels in the pre- and post-periods were 2.4 and 1.2 ng/dL, respectively (*p* = 0.028). Besides these two variables, the pre- and post-periods showed no significant differences in male sex, Charlson comorbidity index score, KTAS, qSOFA score criteria, pre-ED antibiotics, CRP, lactate levels, septic shock, SOFA score, length of hospital stay, and length of ICU stay. The most common sources of infection were the respiratory and genitourinary systems in both groups.

### 3.2. Outcomes

The outcomes are outlined in Table 2. Overall compliance with the SSC guidelines’ bundle was significantly higher in the post-period group than in the pre-period group (10.8% vs. 54.6%; *p* < 0.001). The following items also individually showed significantly higher rates in the post-period: appropriate fluid resuscitation, 77.5% vs. 89.2% (*p* < 0.001); blood culture before antibiotics, 80.7% vs. 96.5% (*p* < 0.001); and at least two lactate measurements within 6 h of ED presentation, 11.7% vs. 84.1% (*p* < 0.001). However, there was no significant difference in the rate of broad-spectrum antibiotic administration within 3 h of ED presentation (71.5% vs. 75.9%; *p* = 0.214). The post-period showed significantly lower 30-day mortality than pre-period did (pre: 37.3% vs. post: 29.5%; *p* = 0.037). There was no significant difference in 7- and 14-day mortality between the two groups. Furthermore, there was no significant difference in time to first antibiotic administration (125 vs. 121 min; *p* = 0.597).

### 3.3. Risk Factors For 30-Day Mortality 

Among all 631 patients, 211 died within 30 days of ED presentation (Table 2). During the pre-period, 37.3% patients died within 30 days of ED presentation. During the post-period, this proportion decreased to 29.5% (*p* = 0.037). Among the 273 septic shock patients, the 30-day mortality rates were 51.8% and 40.1% in the pre- and post-period groups, respectively (*p* = 0.032).

We constructed Cox proportional hazards models for 30-day mortality (Table 3). In the univariate analysis, the variables with *p* ≤ 0.2 were age, SOFA score, septic shock, overall compliance with SSC guidelines, post-period, CRP, lactate level, procalcitonin level, and time to first antibiotic administration. We included the variables with *p* ≤ 0.2 in the multivariate analysis. Multivariate analysis showed that the risk factors for 30-day mortality were age (Hazard Ratio (HR), 1.013; 95% Confidence Interval (CI), 1.002–1.023; *p* = 0.021), SOFA score (HR, 1.21; 95% CI, 1.15–1.26; *p* = 0.002), and serum lactate level (HR, 1.06; 95% CI, 1.02–1.09; *p* = 0.003). Overall compliance with the SSC guidelines (Table 3) was statistically significant (HR, 0.62; 95% CI, 0.44–0.86; *p* = 0.004).

**Figure 2:**
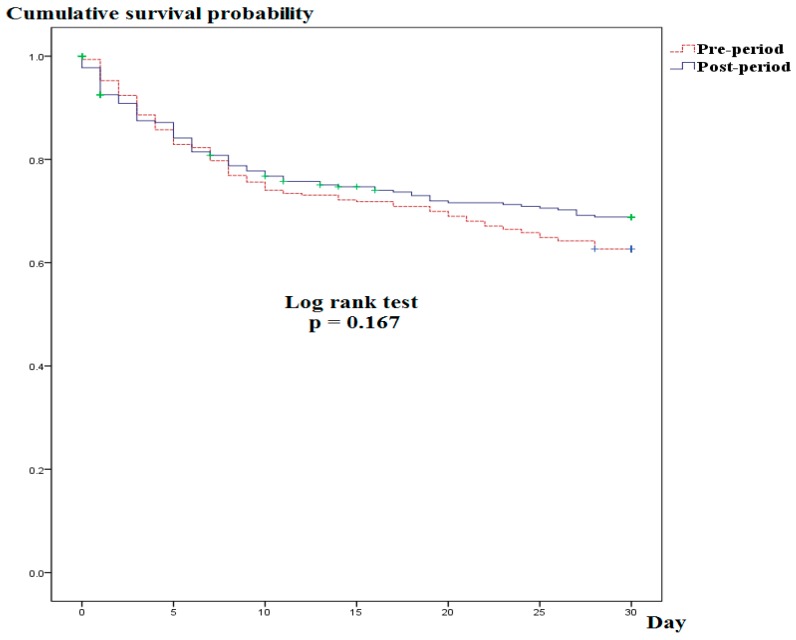
Kaplan–Meier curve for 30-day mortality stratified by study period (pre- vs post-period).

Figure 2 shows the Kaplan–Meier curves for 30-day mortality stratified by study period (pre- vs post-period). The survival curves did not differ between the periods (log-rank test, *p* = 0.167).

### 3.4. Comparisons Between the Compliance and Non-Compliance Groups 

The baseline characteristics of the compliance and non-compliance groups are summarized in Table 4. The compliance group (*n* = 206) included patients treated with overall SSC bundle compliance while in the ED. The non-compliance group (*n* = 425) included patients who were not treated via SSC bundle compliance. No significant differences in age, sex ratio, Charlson comorbidity index, C-reactive protein, procalcitonin, lactate, septic shock, or SOFA score were observed between the groups. The 30-day mortality rate was significantly lower in the compliance group than that in the non-compliance group (27.2% vs. 36.5%, *p* = 0.020).

We performed univariate and multivariate logistic regression of the overall population (*n* = 631) to identify covariates for predicting compliance with SSC guidelines (Table 5). In univariate logistic regression, only the post-period was significant in predicting overall compliance with the SSC guidelines (OR, 9.98; 95% CI, 6.56–15.17; *p* < 0.001). In multivariate logistic regression, the post-period was a predictive factor for overall compliance with the SSC guidelines. 

**Figure 3:**
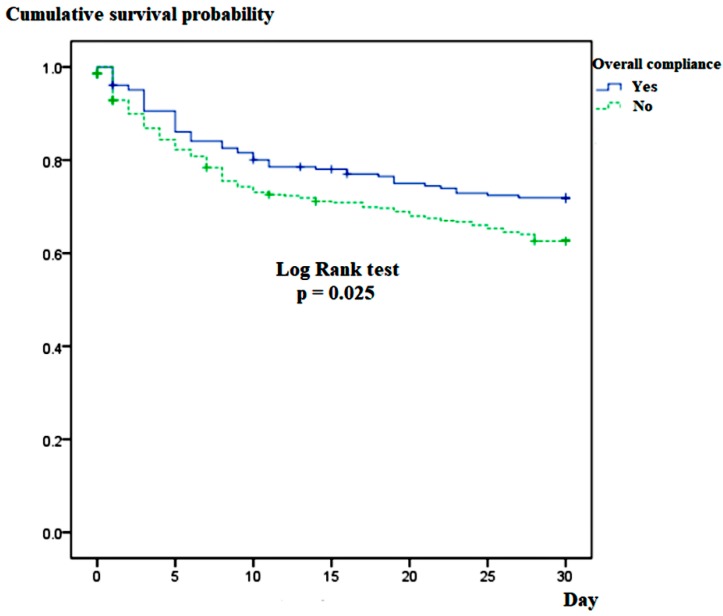
Kaplan–Meier curves for 30-day mortality stratified by overall compliance with surviving sepsis campaign guidelines.

Figure 3 shows the Kaplan–Meier curves for 30-day mortality stratified by SSC guideline compliance. The compliance group showed a significantly lower 30-day mortality rate than the non-compliance group (log-rank test, *p* = 0.025).

## 4. Discussion

The i-SMS implementation improved SSC guideline compliance among patients with sepsis and septic shock as defined by Sepsis-3. Despite significant differences in the 30-day mortality between pre- and post-periods, we could not verify the causal effect of the program on 30-day mortality reduction. Age, SOFA score, serum lactate level, and overall compliance with SSC guidelines were associated factors for 30-day mortality.

Delayed application of the specific three-hour bundle recommended in the SSC guidelines adversely impacted outcomes, while applying the guidelines with shorter delays was associated with better outcomes [19]. Concordant with a previous study, our study showed that overall compliance with the SSC bundle may be a protective factor against 30-day mortality. A systemic review showed that computerized clinical decision support systems improved practitioner performance [20]. A real-time sepsis alert system increased early therapeutic and diagnostic interventions among non-ICU patients at risk for sepsis [16]. The study evaluated whether the implementation of an automated sepsis screening and alert system facilitated early appropriate interventions. A real-time sepsis alert was implemented for the intervention group, which notified the charge nurse on the patient’s hospital ward by text page. Antibiotic escalation, intravenous fluid administration, oxygen therapy, and diagnostic tests were all increased in the intervention group. Moreover, an electronic surveillance system combined with multi-focal interventions for the early detection of sepsis improved survival outcomes among sepsis patients [21].

However, real-time modified systemic inflammatory response syndrome (SIRS) criteria alerts sent to physicians in a medical ICU did not influence therapeutic interventions for sepsis or significantly alter patient outcomes [22]. Another study showed that an electronic tool for sepsis evaluation and management did not influence SSC bundle compliance and outcomes, possibly due to low utilization [23]. In that study, patients were randomized to usual care or to a group with an available electronic tool capable of importing, synthesizing, and displaying sepsis-related data from medical records that used logic rules to offer individualized evaluations of sepsis severity and response to therapy. The authors reported no difference between the two groups regarding the primary outcome of time to completion of all indicated SSC 6-hour bundle elements or time to completion of each element individually. Furthermore, intensive care unit (ICU) mortality, ICU-free days, and ventilator-free days also did not differ between groups. The discrepancies between the results of prior studies likely resulted from differences in individual electronic systems, study population characteristics, disease definitions, or system utilization rates. In the present study, compliance with the SSC guidelines bundle significantly improved after implementing the i-SMS. Furthermore, compliance also may be protective against 30-day mortality. Although the post-period was not a protective factor for 30-day mortality in the Cox proportional hazards model, implementation of the i-SMS might have had secondary effects on survival outcome by improving SSC guideline compliance.

Although the qSOFA score was not developed to replace current ED sepsis screening tools [24], we used qSOFA criteria to screen for sepsis in the i-SMS. Sepsis-3 definitions recommend that the qSOFA score be used to identify sepsis patients with poor outcomes outside the ICU [6,24]. Among patients admitted from the ED with a diagnosis of an infectious disease, the qSOFA criteria performed as well or better than SIRS criteria, severe sepsis criteria, or lactate levels in predicting critical illness [25]. Furthermore, another study proposed that the qSOFA should replace SIRS as the screening tool for sepsis [26]. However, a different study showed poor qSOFA performance as a screening tool for the early identification of sepsis in the ED [27]. Although the qSOFA score has a lower sensitivity for detecting sepsis compared to that for SIRS, it performs better than SIRS in predicting critical illness [25]. Therefore, the qSOFA score is a valuable screening tool for identifying sepsis patients with an increased risk of mortality. In a recent study using in-hospital mortality, repeated qSOFA measurements showed a greater predictive ability for sepsis compared with a single measurement [17].

Three large prospective randomized controlled trials (ARISE, ProCess, and ProMISe) have been performed among septic shock patients. The trials revealed no significant difference in mortality between the early goal-directed therapy (EGDT) and the usual ED care [28,29,30]. Based on these findings, the 2016 SSC guidelines no longer recommend EGDT. Furthermore, a recent meta-analysis reported that EGDT did not result in better outcomes than usual care and was associated with higher hospitalization costs across a broad range of patient and hospital characteristics [31]. Therefore, we did not include EGDT in the i-SMS.

Our study has several limitations. First, this was a pre-post study; thus, it was inevitably prone to selection bias, introduction of confounders, and uneven clinical characteristics between the two groups. Fortunately, the pre- and post-period groups showed similar characteristics and disease severity. Therefore, propensity score matching was unnecessary in the present study. Second, the method of enrollment differed between the two groups. To minimize possible bias, we used the same clinical pathway for both groups. Third, this was a single-center study conducted at a tertiary teaching hospital. The study population was composed largely of older adults with various comorbidities. The intervention effect might differ in a multicenter study or a study based on a younger population with fewer comorbidities. Fourth, we included sepsis patients with initial qSOFA scores ≥2. The qSOFA score showed only moderate sensitivity for detecting sepsis [27]; therefore, sepsis patients with initial qSOFA scores <2 may have been included. Finally, the post-period group results might have been influenced by the Hawthorne effect. The awareness that staff performance and patient outcomes were being monitored might have altered the activity and alertness of ED physicians and nurses.

## 5. Conclusions

After implementation of the i-SMS in ED, compliance with the SSC guidelines improved and all-cause 30-day mortality decreased among patients with sepsis and septic shock as defined by Sepsis-3. However, we could not verify the causal effect of the i-SMS on the 30-day mortality reduction.

## Figures and Tables

**Table 1:** Baseline characteristics of the study population.

Characteristic	Pre-Period (*n* = 316)	Post-Period (*n* = 315)	*p*-Value
Age (years), median (IQR)	74 (64–81)	77 (65–83)	0.048
Age class (years), *n* (%)			
<50	25 (8)	24 (8)	
50–59	78 (25)	76 (24)	
60–69	103 (33)	105 (33)	
70–79	71 (22)	72 (23)	
≥80	39 (12)	38 (12)	
Male, *n* (%)	187 (59.2)	164 (52.1)	0.083
Charlson comorbidity index, median (IQR)	4 (3–6)	4 (3–5)	0.546
Korean triage acuity scale, mean (SD)	2.3 (0.6)	2.2 (0.6)	0.627
Quick SOFA criteria, *n* (%)			
RR ≥ 22/min	147 (47)	144 (46)	0.718
SBP ≤ 100 mmHg	132 (42)	137 (43)	0.752
Altered mental status (GCS < 15)	159 (50)	156 (50)	0.879
Pre-ED antibiotics, *n* (%) (≤12 h)	31 (10)	33 (10)	0.317
Infection sites, *n* (%) (multiple selections, if any)			
Respiratory	198 (63)	201 (64)	0.734
Genitourinary	75 (24)	71 (23)	0.697
Gastrointestinal	36 (11)	34 (11)	
Skin and soft tissue	11 (3)	12 (4)	
Other sites	21 (7)	19 (6)	
Biomarkers, median (IQR)			
CRP (mg/dL)	10.5 (4.6–18.3)	10.6 (4.7–18.5)	0.734
Procalcitonin (ng/ml)	2.4 (1.3–11.8)	1.2 (0.8–9.7)	0.028
Lactate (mmol/L)	2.9 (1.2–5.1)	2.8 (1.1–4.9)	0.821
Septic shock, *n* (%)	135 (42.7)	138 (43.8)	0.779
SOFA score, median (IQR)	8 (4–11)	8 (5–11)	0.343
Length of hospital stay (days), median (IQR)	10 (3–15)	9 (3–14)	0.296
Length of ICU stay (days), median (IQR)	5 (2–7)	4 (2–6)	0.213

IQR = interquartile range, SD = standard deviation, SOFA = sequential organ failure assessment, RR = respiratory rate, SBP = systolic blood pressure, GCS = Glasgow coma scale, ED = emergency department, CRP = C-reactive protein, ICU = intensive care unit.

**Table 2:** Pre- and post-period outcomes among sepsis and septic shock patients.

Outcomes	Pre-Period (*n* = 316)	Post-Period (*n* = 315)	*p*-Value
Overall compliance with SSC bundle, *n* (%)	34 (10.8)	172 (54.6)	<0.001
Appropriate fluid resuscitation	245 (77.5)	281 (89.2)	<0.001
Broad-spectrum antibiotics administered within 3 h of ED presentation	226 (71.5)	239 (75.9)	0.214
Blood culture before antibiotic administration	255 (80.7)	304 (96.5)	<0.001
At least two lactate level measurements within 6 h of ED presentation	37 (11.7)	265 (84.1)	<0.001
Time to first antibiotic administration (min), median (IQR)	125 (79–203)	121 (75–198)	0.597
All-cause 7-day mortality, *n* (%)	64 (20.3)	58 (18.4)	0.558
All-cause 14-day mortality, *n* (%)	87 (27.5)	76 (24.1)	0.329
All-cause 30-day mortality, *n* (%)	118 (37.3)	93 (29.5)	0.037

SSC = Surviving Sepsis Campaign, ED = emergency department, IQR = interquartile range.

**Table 3:** Risk factors for 30-day all-cause mortality in univariate and multivariate Cox proportional hazards models.

Variable	Hazards Ratio (95% CI)	*p* Value	Adjusted Hazards Ratio (95% CI)	*p* Value
Age	1.02 (1.01–1.03)	0.001	1.013 (1.002–1.023)	0.021
Male	1.08 (0.83–1.42)	0.564		
SOFA score	1.27 (1.22–1.32)	<0.001	1.21 (1.15–1.26)	0.002
Septic shock	3.45 (2.58–4.60)	<0.001	1.83 (0.97–2.76)	0.18
Overall compliance with SSC bundle	0.71 (0.52–0.96)	0.027	0.62 (0.44–0.86)	0.004
CRP	1.01 (0.997–1.022)	0.144	1.02 (0.995–1.028)	0.168
Lactate	1.09 (1.06–1.12)	<0.001	1.06 (1.02–1.09)	0.003
Procalcitonin	1.004 (1.00–1.01)	0.053	1.001 (0.997–1.006)	0.779
Time to first antibiotics (min)	0.999 (0.998–1.000)	0.041	0.998 (0.997–1.000)	0.059
Post-period	0.83 (0.63–1.09)	0.172	0.75 (0.55–1.04)	0.151

CI = confidence interval, SOFA = sequential organ failure assessment, SSC = surviving sepsis campaign, CRP = C-reactive protein.

**Table 4:** Characteristics and short-term mortality in the overall and non-compliance groups.

Characteristic	Overall Compliance Group (*n* = 206)	Non-Compliance Group (*n* = 425)	*p*-Value
Age (years), median (IQR)	75 (63–82)	76 (66–83)	0.794
Male, *n* (%)	107 (51.9)	244 (57.4)	0.195
Charlson Comorbidity Index, median (IQR)	4 (3–5)	4 (3–6)	0.631
Biomarkers, median (IQR)			
CRP (mg/dL)	10.5 (5.3–18.4)	10.5 (5.4–18.5)	0.867
Procalcitonin (ng/mL)	1.0 (0.3–8.5)	1.8 (0.4–14.7)	0.148
Lactate (mmol/L)	2.9 (1.5–5.1)	2.9 (1.4–4.9)	0.484
Septic shock, *n* (%)	93 (45.1)	180 (42.4)	0.507
SOFA score, *n* (%)	8 (6–10)	8 (5–10)	0.111
Post-period, *n* (%)	172 (83.5)	143 (33.6)	<0.001
7-day mortality, *n* (%)	32 (15.5)	90 (21.2)	0.092
14-day mortality, *n* (%)	44 (21.4)	119 (28.0)	0.074
30-day mortality, *n* (%)	56 (27.2)	155 (36.5)	0.020

IQR = interquartile range, CRP = C-reactive protein, SOFA = sequential organ failure assessment.

**Table 5:** Predictive factors for compliance with the Surviving Sepsis Campaign guidelines in univariate and multivariate logistic regression analysis.

Variable	Odds Ratio (95% CI)	*p*-Value	Adjusted Odds Ratio (95% CI)	*p*-Value
Age	1.23 (0.88–1.60)	0.472		
Male	1.06 (0.88–1.24)	0.374		
SOFA score	0.94 (0.90–1.13)	0.398		
Septic shock	0.89 (0.64–1.25)	0.507		
CRP	1.04 (0.97–1.18)	0.514		
Lactate	0.93 (0.86–1.02)	0.089	0.94 (0.83–1.12)	0.207
Procalcitonin	1.04 (0.93–1.13)	0.495		
Post-period	9.98 (6.56–15.17)	<0.001	9.51 (6.38–14.03)	<0.001

CI = confidence interval, SOFA = sequential organ failure assessment, CRP = C-reactive protein.
